# Detecting Grapevine Virus Infections in Red and White Winegrape Canopies Using Proximal Hyperspectral Sensing

**DOI:** 10.3390/s23052851

**Published:** 2023-03-06

**Authors:** Yeniu Mickey Wang, Bertram Ostendorf, Vinay Pagay

**Affiliations:** 1 School of Agriculture, Food & Wine, Waite Research Institute, The University of Adelaide, PMB 1, Glen Osmond, SA 5064, Australia; 2 CSIRO Manufacturing, 13 Kintore Ave, Adelaide, SA 5000, Australia; 3 School of Biological Sciences, The University of Adelaide, Molecular Life Sciences Building, North Terrace Campus, Adelaide, SA 5005, Australia

**Keywords:** GLRaV-1, GVA, GLD, disease detection, proximal sensing, spectroradiometer, PLS-DA

## Abstract

Grapevine virus-associated disease such as grapevine leafroll disease (GLD) affects grapevine health worldwide. Current diagnostic methods are either highly costly (laboratory-based diagnostics) or can be unreliable (visual assessments). Hyperspectral sensing technology is capable of measuring leaf reflectance spectra that can be used for the non-destructive and rapid detection of plant diseases. The present study used proximal hyperspectral sensing to detect virus infection in Pinot Noir (red-berried winegrape cultivar) and Chardonnay (white-berried winegrape cultivar) grapevines. Spectral data were collected throughout the grape growing season at six timepoints per cultivar. Partial least squares-discriminant analysis (PLS-DA) was used to build a predictive model of the presence or absence of GLD. The temporal change of canopy spectral reflectance showed that the harvest timepoint had the best prediction result. Prediction accuracies of 96% and 76% were achieved for Pinot Noir and Chardonnay, respectively. Our results provide valuable information on the optimal time for GLD detection. This hyperspectral method can also be deployed on mobile platforms including ground-based vehicles and unmanned aerial vehicles (UAV) for large-scale disease surveillance in vineyards.

## 1. Introduction

Virus-associated disease such as grapevine leafroll disease (GLD) affects grapevine health worldwide [1]. GLD has native impacts on both grape quality and yield, for example, lower anthocyanin accumulation rate resulting in poor colour development in red berries, fewer total soluble solids leading to lower °Brix, and smaller cluster size resulting in low yield [1,2,3,4]. This viral disease is vectored by sap-feeding insects including mealybugs and soft scales in vineyards [5,6] and causes long-term economic loss if left uncontrolled [4,7,8].

Grapevine leafroll-associated viruses (GLRaVs) such as GLRaV-1, -2, -3, -4, and -7 cause GLD in grapevines [9]. In Australian vineyards, GLRaV-1, -3, and -4 have been frequently found [10]. Co-infection could also happen in an individual vine such as infection with multiple GLRaVs (e.g., GLRaV-1 and GLRaV-3), different strains or variants of one GLRaV, or with multiple grapevine viruses. As an example of the latter, grapevine virus A (GVA) is often found to co-exist with GLRaV in vines, as GLRaVs act as a helper virus for GVA transmission by insect vectors [11]. In certain cultivars including Shiraz, Merlot, and Malbec, the co-infection of GLRaVs and GVA could lead to another devastating disease—Shiraz disease [12].

As there is no cure for virus-infected vines, preventive disease management methods such as quarantine, pest control, and roguing infected vines are the only effective methods to control viral diseases in vineyards [13,14]. However, roguing requires accurate diagnostic results for guidance. Laboratory-based diagnostic methods including the serological method—enzyme-linked immunosorbent assay (ELISA)—and molecular method—reverse transcription polymerase chain reaction (RT-PCR)—often provide accurate results for virus detection; however, testing rates are hampered by their high costs. Visual assessment is a phenotypic detection method relying on the disease symptoms, which can be an alternative detection method and relatively cost-effective. For example, GLD in red-berried cultivars shows symptoms of reddening on leaves with green veins and rolling leaf edges, while in white cultivars, the same virus shows symptoms of leaf rolling at the edge, and subtle leaf rolling, which is not consistent and more difficult to detect visually compared to red-berried cultivars [15]. Thus, symptom-based detection of GLD is only relatively reliable for red cultivars and for specific leafroll viruses such as GLRaV-3 [16]. The variable reliability of the visual method stems from the varying levels of experience of the surveyors [17].

Hyperspectral technology has the proven capability of detecting plant diseases and, in some cases, even on asymptomatic leaves, as it measures spectra beyond those of human vision [18]. Various studies have used the proximal hyperspectral sensing method for GLD detection directly in contact with grapevine leaves or under a controlled environment in a laboratory [19,20,21,22,23]. However, these studies used active sensors, which rely on artificial illumination [24]. The benefit of active sensors is their consistent incident light levels and spectra that are unaffected by the external light environment. The drawback of using these types of sensors is that they can be time-consuming, as only a small portion of single leaves are measured in the field or leaf samples have to be taken to the laboratory for measurements. To address the speed limit of traditional proximal hyperspectral sensors, Bendel et al. [25] used an over-the-row grape harvester equipped with an active hyperspectral sensor for GLD detection in a vineyard. This method of detection was relatively quick; however, the setup of the system was complicated and impractical for growers to use on a routine basis without significant technical expertise.

In contrast to active sensors, passive sensors use natural light (solar radiation) for the illumination of leaves to measure their reflectance spectra. The detection area is generally larger in size depending on the measure distance, and the measurement can be relatively fast and simple to handle in practice [26]. However, very few studies have used passive hyperspectral sensors to detect grapevine viral disease at a canopy scale. Nguyen et al. [27] used a proximal sensing hyperspectral camera—Specim IQ—to detect grapevine vein clearing virus (GVCV) infections; however, the camera was a line scanner imaging sensor that required a fixed position and ca. 0.5 min for each hyperspectral image collection, which was time-consuming.

Various machine-learning algorithms have been used for disease classification in hyperspectral data, such as partial least squares (PLS), support vector machines, random forests, convolutional neural networks, and more recently deep learning algorithms [27,28,29]. PLS is the gold standard for binary classification; it is computationally inexpensive compared to other algorithms [30]. The principle of PLS is similar to principle component analysis, but it is a supervised method that uses the known information as input to train the model [31]. PLS relates two data matrices X (spectral dataset for all samples) and Y (disease status for all samples) to maximise the co-variance between components from the two data sets [32,33]. The output of PLS analysis is a linear model that is used for discrimination problems, also called partial least squares-discriminant analysis (PLS-DA) [34]. Various studies have used the PLD-DA method to analyse spectral data for plant disease classification [35,36,37,38,39].

The present study used a hand-held passive spectroradiometer for the proximal detection of virus infection in grapevines, and PLS-DA was used for modelling. Our aim was to (1) evaluate the potential of passive proximal hyperspectral sensors to detect virus infection on both red- and white-berried grape canopies in field conditions; (2) optimise the hyperspectral data processing workflow to produce a robust modelling method for disease prediction using the PLS-DA algorithm; and (3) determine the optimal growth stage for disease detection using this approach. This study could provide valuable guidance for using proximal sensing such as detection time, measuring direction, appropriate environmental conditions, and measurement distance. Moreover, this method is readily applicable to both ground-based mobile platforms as well as low-altitude remote sensing platforms such as unmanned aerial vehicles (UAV) and airplanes for large-area disease surveillance.

## 2. Materials and Methods

### 2.1. Experimental Site and Plant Virus Testing

This study was conducted in a commercial vineyard located in the Adelaide Hills wine region—Kuitpo, SA, Australia (35°13′ S, 138°39′ E). Spectral data collection and plant tissue sampling were performed in the growing season 2020/21, from September 2020 to April 2021. Two grape cultivars—Pinot Noir (red cultivar) and Chardonnay (white cultivar) were selected for the study. Both cultivars were planted in 1988, own-rooted, on a podsol soil type. The vineyard was drip irrigated at 1.8 ML per hectare per season. Vines were spur pruned to 26 buds per metre, and the canopy was uniform in size in the block. Two sulphur sprays per season to control the pest were based on recommendations by a local viticulture consultant. The grapevines did not have any visible symptoms of nutrient deficiencies or diseases other than GLD. A ground-based visual inspection for GLD symptoms (leaf reddening on Pinot Noir and leaf rolling on Chardonnay) was conducted during the previous growing season, 2019/20, which showed a ca. 40% infection rate in the Pinot Noir block, and ca. 60% infection rate in the adjacent Chardonnay block.

Two rows of vines of each grape cultivar were selected for measurement for a total of 173 Pinot Noir and 174 Chardonnay vines. Each vine was tested with ELISA for ground truthing (presence or absence of leafroll virus). The leaf petiole tissue was sampled as suggested by Monis and Bestwick [40] at the harvest stage (March 2021). Virus testing was conducted with the DAS-ELISA (double antibody sandwich-enzyme-linked immunosorbent assay) test kits produced by Bioreba (Reinach, Switzerland). The test method strictly followed the procedure produced by Bioreba [41]. Four viruses (GLRaV-1, -3, -4, and GVA) were tested for each sample as per the manufacturer’s instructions. To confirm the accuracy of ELISA results, 10 of the ELISA-tested positive samples and 20 negative samples were tested with RT-PCR (GLRaV-1, -3, -4, -4 strain 6, -4 strain 9, and GVA) in a NATA-accredited commercial laboratory—Affinity Labs (Adelaide, SA, Australia). The RT-PCR results matched all ELISA-tested results. Based on the test results, the samples were grouped into two classes: disease (GLRaV-1 + GVA positive) and healthy (tested negative for GLRaV-1, -3, -4, and GVA).

### 2.2. Spectral Data Collection

**Figure 1:**
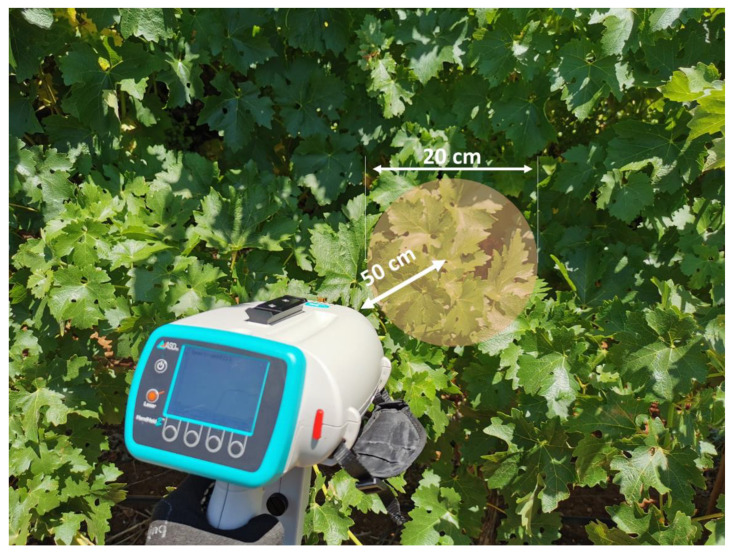
Spectral data measurement. The yellow shaded area shows the circular field of view of diameter of approx. 20 cm based on the approx. 0.5 m horizontal measurement distance.

Leaf spectral reflectance data were collected using a portable hand-held spectroradiometer (ASD FieldSpec^®^ HandHeld 2, Malvern Panalytical Ltd., Malvern, UK). The instrument is a silicon array-based sensor that measures light spectra between 325–1075 nm at 1 nm spectral resolution. The optical input has a 25° conical field of view (FOV). Data collection was conducted under sunny conditions at each timepoint. A Labsphere Spectralon^®^ white reference panel (Halma plc, Amersham, UK) was used as the calibration target. The spectroradiometer was calibrated with the white reference (reflectance = 1) immediately before the measurement and was re-calibrated every 10 min to account for the changing illumination due to the sun angle. The instrument was held horizontally (parallel to the ground plane) and pointed to the centre of the canopy, perpendicular to the vertical canopy wall, as shown in Figure 1. The measurement distance was approx. 0.5 m from the canopy, which represented a ca. 20 cm diameter circle on the canopy (approx. three to four fully expanded and mature leaves).

Since the measurement used solar radiation as the light source, consistency in incident light was critical. Different instrument positions relative to the canopy were tested at the beginning of the season, including holding the sensor vertically and horizontally to measure the top and side of the canopy as well as the sunlit and sunshade sides of the canopy. After several test measurements, we determined that the horizontal position (perpendicular to the canopy wall) at the sunlit side of the canopy provided the most consistent results. All vines in each of the two rows were measured within the time window of 12:00–14:00 h. One spectrum measurement per vine was collected at a monthly interval between the months of November and April. The measurements started from the flowering development stage (EL-23) in November until post-harvest (April), which resulted in six timepoints.

### 2.3. Data Processing and Modelling

The spectral data processing and modelling were performed as described below using the PLS_Toolbox software plugin (v.9.0, Eigenvector Research, Inc., Manson, WA, USA) within the MATLAB R2021b (The MathWorks Inc., Natick, MA, USA) software environment.

#### 2.3.1. Spectral Data Pre-Processing

To compensate for variations in the leaf angle, sun angle, and instrument holding position during field measurement, the raw spectral data was pre-processed. Pre-processing allows for noise removal, light scattering correction, spectral deviation compensation, and scale optimisation [42,43]. The transformed spectral data improved the prediction model and increased robustness.

**Figure 2:**
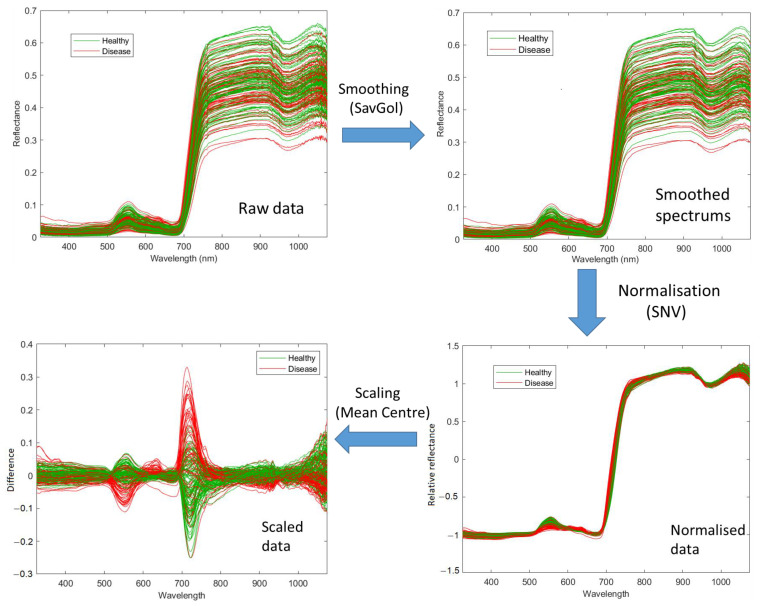
Spectral data pre-processing steps for Pinot Noir at the February 2021 timepoint. The raw spectral data is smoothed using the SavGol algorithm with seven bandwidths, then normalised using the standard normal variate algorithm and, lastly, scaled using the Mean Centring algorithm for 750 bands.

Pre-processing in this study included three steps: smoothing, normalisation, and scaling. Firstly, the raw data were smoothed using a Savitzky-Golay Filter (SavGol) [44] with the filter width w = 7. The high-frequency noise was smoothed from the raw data, especially for the wavelengths below 400 nm and above 900 nm, due to the sensitivity of the sensor being lower in these regions. Secondly, the smoothed spectral data were normalised with the standard normal variate (SNV) method [45]. The normalised data overcomes multiplicative and baseline effects caused by a difference in leaf angles, measurement distance and angles. Thirdly, the data were scaled using the mean centre method [46], which brought all wavelengths to the same magnitude and improved the models in the current study. Figure 2 demonstrates an example of pre-processing for 173 spectral data derived from Pinot Noir vines.

#### 2.3.2. Outlier Removal

The grapevine canopy wall sometimes has gaps and holes that can cause outliers in the dataset. An outlier removal step is necessary, as the outliers could significantly influence the model when the sample number is not large. The Hotelling’s T^2^ and Q residuals plot was used for outlier identification [47]. The abnormally high values in either Hotelling’s T^2^ or Q residuals were considered outliers and were removed from the dataset; for example, either the Hotelling’s T^2^ value or Q residual values larger than 10 were considered outliers in the data set, which resulted in two outliers being removed from the April 2021 timepoint from the Chardonnay data set and one outlier being removed from the April 2021 timepoint from the Pinot Noir data set.

#### 2.3.3. Cross-Validation

Cross-validation (CV) is a practical method for assessing the performance of the model, particularly for small sample sizes such as in this study [48]. The Venetian blinds method was used for the CV method [49]. The data was split into 10 divisions (blinds), and one sample per blind was taken for validation. Thus, the model was built with 90% of the data and validated with 10% of the data. The process was repeated 10 times to cover all data in the training data.

#### 2.3.4. Modelling

PLS-DA was used for the classification modelling in this study due to its simplicity and its computationally efficient and interpretable results [50]. Similar to the components in principle component analysis, latent variables (LVs) represent the linear combination of variables in PLS-DA models [51]. The number of LVs selected for the PLS-DA model could affect the performance of the model. Typically, increasing the number of LVs in a model results in lower prediction error (Equation (1)). However, redundant LVs could result in overfitting, thus the CV error was considered in determining the appropriate number of LVs to use in the model. The sum of error in CV was calculated for selected LVs; this procedure is known as Wold’s R criterion [52]. To build a robust model, additional LVs should be selected only when the CV error decreases significantly, which may not necessarily occur at the lowest error. A scree plot of the calibration and CV errors aids the LVs selection.

(1) $$\mathrm{Error}=\frac{\mathrm{FP}+\mathrm{FN}}{\mathrm{FP}+\mathrm{FN}+\mathrm{TP}+\mathrm{TN}}$$

where FP is the number of false positives (healthy that were incorrectly classified disease), FN is the number of false negatives (disease that was incorrectly classified as healthy), TP is the number of true positives (disease that was correctly identified), and TN is the number of true negatives (healthy that were classified correctly).

**Figure 3:**
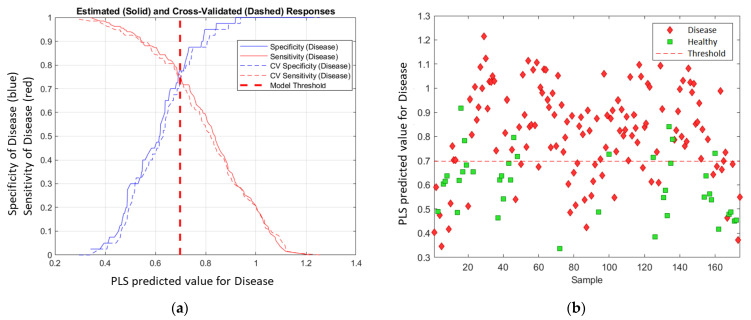
PLS-DA threshold determination. (**a**) ROC curve for disease class; (**b**) plot of PLS predicted value for disease class for Chardonnay in March 2021. The threshold was 0.698 in this model.

The PLS-DA model used LVs to calculate each data point (one spectral measurement) and project the result to a linear model—PLS predicted value (disease). The higher the value, the more likely the sample was to be in the predicted (disease) class. Then, a threshold was calculated to discriminate between the two classes. The threshold was based on a balance between sensitivity (Equation (2)) and specificity (Equation (3)) to minimise the error. The receiver operating characteristic (ROC) curve was used to determine the threshold, and the PLS-predicted value at the intersection of sensitivity and specificity was selected as the threshold (Figure 3a). Figure 3b visually illustrates the prediction for the Chardonnay model at the March 2021 timepoint. The samples that lie above the threshold were predicted as ‘Disease’ while samples below the threshold were predicted as ‘Healthy’.

(2) $$\mathrm{Sensitivity}=\frac{\mathrm{TP}}{\mathrm{TP}+\mathrm{FN}}$$

(3) $$\mathrm{Specificity}=\frac{\mathrm{TN}}{\mathrm{TN}+\mathrm{FP}}$$

The PLA-DA models were developed for each cultivar from spectral data collected at each timepoint. The performance of each model was evaluated based on the binary confusion matrix of calibration and CV results (TP, TN, FP, and FN); the sensitivity and F1 score (Equation (4)); the accuracy (Equation (5)), and Matthews correlation coefficient (MCC) [53] (Equation (6)).

(4) $$F1\;\mathrm{score}=\frac{2\cdot \mathrm{TP}}{2\cdot \mathrm{TP}+\mathrm{FP}+\mathrm{FN}}$$

(5) $$\mathrm{Accuracy}=\frac{\mathrm{TP}+\mathrm{TN}}{\mathrm{TP}+\mathrm{TN}+\mathrm{FP}+\mathrm{FN}}=1-\mathrm{Error}$$

(6) $$\mathrm{MCC}=\frac{\mathrm{TP}\cdot \mathrm{TN}-\mathrm{FP}\cdot \mathrm{FN}}{\sqrt{\left(\mathrm{TP}+\mathrm{FP}\right)\cdot \left(\mathrm{TP}+\mathrm{FN}\right)\cdot \left(\mathrm{TN}+\mathrm{FP}\right)\cdot \left(\mathrm{TN}+\mathrm{FN}\right)}}$$

A regression vector plot for each model was generated to identify the important wavelengths required for modelling. Lastly, each model was used for predicting the data from other timepoints for each cultivar.

## 3. Results

### 3.1. Virus Test Results

The laboratory test results showed that all of the virus-infected vines were co-infected with two viruses, GLRaV-1 and GVA, for which 206 out of 347 samples tested positive. All vines tested negative for GLRaV-3 and -4. In addition, 141 vines tested negative for each of the viruses (GLRaV-1, -3, -4, and GVA). The sample sizes of each group for Chardonnay and Pinot Noir are shown in Table 1.

### 3.2. Disease Symptomology

**Figure 4:**
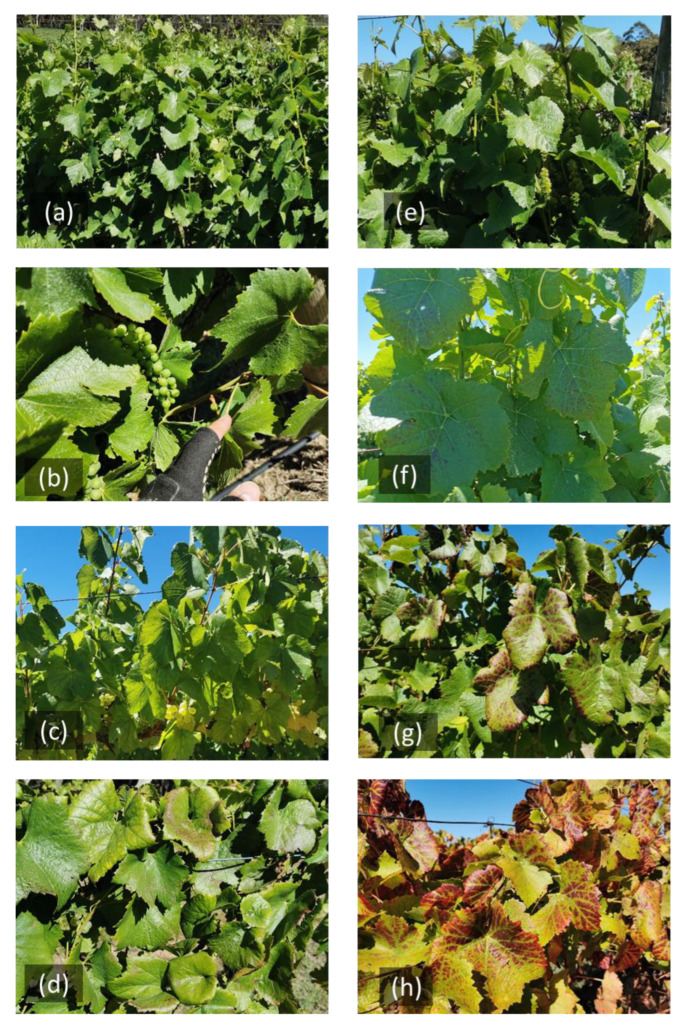
The GLD-infected vines at different development stages. Chardonnay in (**a**) November—flowering stage (EL-17); (**b**) December— pea-size berries; (**c**) February—veraison; (**d**) April—post-harvest. Pinot Noir in (**e**) November—flowering; (**f**) December—pea-size berries; (**g**) February—veraison; (**h**) April—post-harvest.

The disease symptoms for Chardonnay were difficult to visualise. There was no visual difference between healthy and diseased vines at the early flowering stage (EL-23; Figure 4a) and pea-size berry stage (EL-31; Figure 4b). Leaves of the diseased vines started to show mild yellowing before harvest (Figure 4c). Approx. 5% of leaves in the GLD-infected canopy showed leaf-rolling symptoms (Figure 4d). In Pinot Noir, disease symptoms did not appear before the flowering stage (Figure 4e). A few reddening spots appeared on mature leaves after the fruit setting stage (EL-27; Figure 4f). The red leaf and green veins became obvious at the veraison stage (EL-35; Figure 4g). The red leaf disease symptoms were most evident from harvest through post-harvest (Figure 4h).

### 3.3. Critical Spectral Regions for Disease Classification

**Figure 5:**
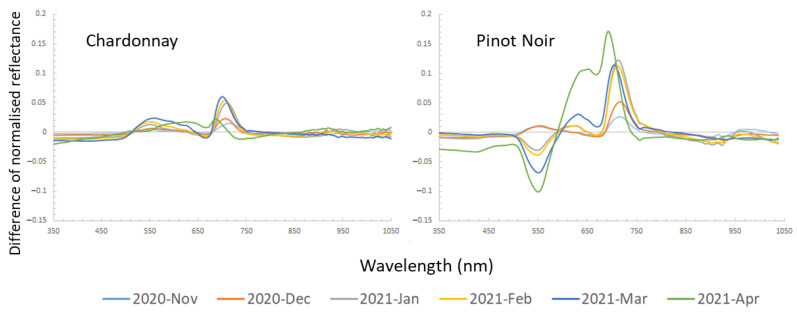
The difference of normalised averaged spectral reflectance of diseased to healthy vines (value at 0) for each timepoint.

The difference between normalised average spectra of diseased and healthy vines was compared at each timepoint for the two cultivars as shown in Figure 5. An overall increase in reflectance difference over time in the diseased vines was observed in Chardonnay, which had the highest relative reflectance in March 2021. However, the difference decreased between the diseased and healthy vines in April. Between 690 to 730 nm in the red-edge region had a higher difference, and between 530 to 630 nm in the visible region showed a small increased reflectance in diseased Chardonnay. This matched the visual symptoms that diseased leaves were slightly yellower than healthy leaves, especially at later growth stages.

In Pinot Noir, the diseased vines showed a similar pattern as Chardonnay in the early season (November and December). However, beginning in January (around the veraison stage), the diseased vine showed a significantly lower reflectance at a peak around 550 nm. Similar to Chardonnay, the red-edge region in Pinot Noir had much higher reflectance compared to healthy vines. In April, the spectral reflectance of diseased vines showed the highest difference to healthy vines compared to other timepoints, which had a higher reflectance at around 650 nm and between 690–730 nm, and lower reflectance at 550 nm.

### 3.4. The Model Results

The comprehensive results, including the confusion matrix, sensitivity, F1-score, accuracy, and MCC for each model, are shown in Table 2 (Chardonnay) and Table 3 (Pinot Noir). In Chardonnay, the model built at the earliest timepoint had the lowest accuracy but gradually improved over time. The best model result was in March, with a sensitivity of 0.76 and F1-score of 0.83 for the disease class; an accuracy of 0.76 and an MCC of 0.47 in the calibration set; and sensitivity and F1-score for the disease class of 0.74 and 0.81, an accuracy of 0.74, and an MCC of 0.41 in the validation set.

In Pinot Noir, the models did not well perform in the early season (November and December). However, the model improved dramatically beginning in January. The accuracy of the model was 0.89 and MCC was 0.78 as the diseased leaf started showing reddening symptoms. The best model was after harvest in April. The sensitivity and F1-score for the disease class were 0.92 and 0.95, respectively, the accuracy was 0.96, and MCC was 0.92 in the calibration set; the sensitivity and F1 score for the disease class were 0.92 and 0.94, respectively, the accuracy was 0.95, and MCC was 0.89 in the validation set.

Due to the unbalanced samples, the F1 score was different between disease and healthy classes in Chardonnay. A low sample number in the healthy class resulted in a low F1 score because it did not count TN.

**Figure 6:**
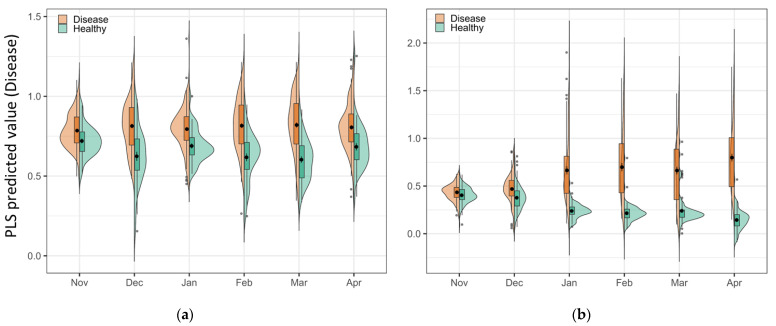
The combined violin plot and box plot of the predicted value of disease from the PLS-DA model for (**a**) Chardonnay, and (**b**) Pinot Noir at each timepoint. The larger the value, the higher the probability of a sample belonging to a diseased vine, and the smaller the value, the lower the probability of a diseased vine. In this binary model, a low value means the sample more likely belongs to a healthy vine. The greater the separation between actual disease and healthy samples, the better the performance of the classification model.

One advantage of the PLS-DA model is the explainable linear relationship between classes [34]. As the spectral signal changes over time, the evidence shows in the PLS predicted value in the combined violin and box plots (Figure 6). The vertical histograms show the sample distribution of both classes in the predicted value axis. Increased separation between healthy and disease classes can be observed over time in the plots. In the Chardonnay plot (Figure 6a), the samples in disease and healthy were close to each other in November and started to separate in December, and the largest separation was in March. In Pinot Noir, two classes overlapped in the first two timepoints; however, a noticeably higher PLS predicted value could be observed in disease samples from January, with the largest difference in April (Figure 6b). This plot matched the visual symptoms and the model results (Table 1 and Table 2) that provide a visual and subjective basis for the model performance.

### 3.5. Model Prediction Matrix

**Figure 7:**
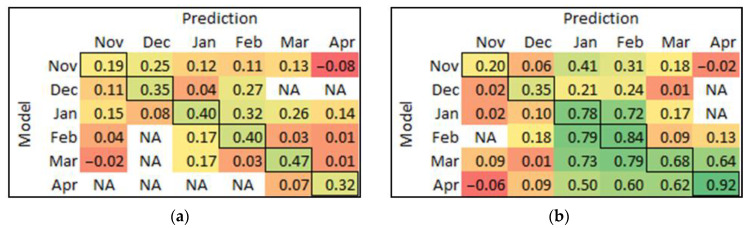
The matrix of MCC from each model prediction of other timepoints for (**a**) Chardonnay, and (**b**) Pinot Noir. The bold cells are the self-predicted results, which are equal to the calibration results. The colour score ranges from red (low value) to yellow (medium) to green (high value). NA means √((TP + FP)∙(TP + FN)∙(TN + FP)∙(TN + FN)) equal to zero that cannot be divided.

The model built at each timepoint was used to predict the data at other times for each cultivar, and MCC was used to assess the prediction performance. MCC can overcome the issue of the imbalanced data set in binary classifications (disease or healthy), which made a better comparison for model performance in both cultivars at the same scale compared to other parameters (accurate, F1-score, and sensitivity) [54]. The matrices of MCC results of the model at each timepoint that were used to predict all other times are presented in Figure 7. In Chardonnay (Figure 7a), only the January model could reasonably predict the data in February; no other models were useful for predictions at different times. In Pinot Noir (Figure 7b), the model built from January data can forward predict February, and February can backward predict January very well. The March model backward predicted both January and February data. The April model had the best forward prediction overall; however, this model did poorly in predicting earlier timepoints.

## 4. Discussion

The current study systematically assessed virus-infected (GLRaV-1 + GVA) vines in both red (Pinot Noir) and white (Chardonnay) winegrape cultivars from flowering to post-harvest stages, both visually as well as with a proximal spectroradiometer, under field conditions. Visual assessments did not allow us to distinguish between virus-infected and healthy vines in the early season (before January in either cultivar). Typical GLD symptoms manifested as reddening on leaves in Pinot Noir and leaf edge rolling in Chardonnay and commenced in the virus-infected vines around late December. The severity of symptoms gradually increased and became visually more evident over time. This indicates that the virus may be multiplying in the vines and increasing the virus concentration or titre level during the growing season [55]. Another study also found there was a higher virus titre in symptomatic vines than in asymptomatic vines [56]. However, the symptomatology of virus infection is complicated and depends on various factors including environment, growth stage, virus isolate, cultivars, and infection ages [15,57]. It is therefore challenging to detect viral diseases with only visual symptomology, especially for the white cultivars, as their symptoms are less visually obvious compared to red cultivars. Montero et al. [58] found the GLRaV-3-infected Malvasia de Banyalbufar grapevine (white cultivar) did not show visual symptoms; however, the leaf net carbon dioxide assimilation and electron transport rates of infected vines were lower compared to healthy vines. This indicates the virus could affect plant growth and physiology even if it is asymptomatic. A systematic investigation of the symptomology of virus-inoculated vines over a period of time for white cultivars should be conducted, similar to the study done on GLRaV-3 infected red cultivars in New Zealand [16,59].

Our study showed that the average spectral reflectance of GLRaV-1 + GVA-infected vines was different to healthy vines in certain spectral regions. In the visible spectrum region (400–700 nm) in Pinot Noir, the green (550 nm) and red regions (630–650 nm) showed a large difference between virus-infected and healthy vines at the later stage, consistent with the findings by Naidu et al. [19], who measured the GLRaV-3-infected leaves in Cabernet Sauvignon and Merlot (both red cultivars) with leaf contact-based spectroradiometer. Gutha et al. [60] demonstrated that the red leaf symptom in GLRaV-3-infected Merlot (red cultivar) was largely caused by the accumulation of anthocyanin in leaves as a response to the stress. The authors also found that infected vines’ leaves contained approx. 20% less chlorophyll and carotenoids than healthy leaves. As anthocyanins mainly absorb green and yellow light, the leaves appear red when anthocyanin content is high [61]. Anthocyanins absorb the spectral wavelength between 500 and 600 nm and especially at 550 nm [62]. This can be observed from January to April in Pinot Noir (Figure 5), a peak low reflectance was around the 550 nm spectral region. As anthocyanin pigments appear red in colour, the red spectral region (620–650 nm) in the virus-infected Pinot Noir had a higher reflectance compared to the healthy vines. In comparison, the virus-infected Chardonnay had poor expression of visual symptoms due to the lack of anthocyanin biosynthesis in white cultivars [63,64]. Thus, it was expected that there would be less difference between healthy and diseased vines in the blue and red spectral regions. We found that virus-infected Chardonnay had a higher average reflectance in the 530–630 nm region, which may be due to lower chlorophyll content in the leaves. In the red-edge region, both virus-infected red and white cultivars showed a high reflectance between 690 and 730 nm, indicating that the plant was under stress. Various studies have shown that the red-edge spectrum has a linear relationship with the chlorophyll content in leaves and is sensitive to stress [65,66,67,68]. This was clearly observed in our study (Figure 5) and suggests that the red-edge spectrum is useful for virus infection detection in both red- and white-berried grapevines. Based on the spectral difference between healthy and diseased vines, a multispectral sensor offers a simpler solution compared to hyperspectral sensing with a red-edge and green bands; e.g., MicaSense RedEdge P and Sentera 6X multispectral cameras could be used for viral disease detection in grapevines.

From the PLS-DA-predicted value plots and the model results, we observed increasing model accuracy over time for both red and white cultivars, which can be related to the development of disease symptoms in grapevines and the associated changes in spectral reflectance. The model prediction results (MCC) matrix (Figure 7b) showed that Pinot Noir models in both January and February could reliably predict each other’s data, indicating that the spectral signatures of virus-infected vines are similar during this period. The best model for Pinot Noir was in April with an accuracy of 0.96 and an MCC of 0.92. This result indicates that the later part of the growing season (post-harvest) is the best time for viral disease detection in red cultivars if using the current proximal sensing method. However, if the vines are machine-harvested, resulting in considerable damage to the canopy, the current method may be unreliable, as the physical damage could change the spectral reflectance of the leaves. In our study, the grapes were hand-harvested with minimum alteration to the canopy, i.e., virtually no damage to the leaves. The accuracy of Chardonnay was low in April; this may be due to the onset of leaf senescence that creates gaps and holes in the canopies, which results in some background noise during the measurement. The best model for virus-infection prediction in Chardonnay was in March (at harvest time) with an accuracy of 0.76 and MCC of 0.47. The result was not considered high compared to Pinot Noir. Future studies could use sensors with different spectral ranges, e.g., shortwave infrared (SWIR). Bendel et al. [25] used a SWIR (1000–2500 nm) hyperspectral sensor to detect viral diseases in grapevines. The authors achieved an accuracy between 0.82 to 0.89 for GLRaV-1 detection, and 0.82 to 1.00 accuracy for GLRaV-3 detection on symptomatic white cultivars (Aligoté, Gewürztraminer, and Silvaner) in the vineyard.

In the present study, only the PLS-DA algorithm was used for modelling, as the purpose of this study was not to compare different statistical and machine learning algorithms, but rather to assess the temporal spectral difference and changes for reliable detection of virus-infected vines. PLS-DA is computationally efficient, relatively fast, and provides easy-to-interpret results that can quickly compare the model results from each timepoint. The model results showed high accuracy in the classification of virus-infected and healthy vines in Pinot Noir and moderate accuracy for Chardonnay. However, various other classification techniques can be used for high dimensional datasets, including linear discriminant analysis, k-nearest neighbours, support vector machine, and artificial neural networks [69]. Many studies compared different modelling techniques for plant disease detection [25,27,70,71]. It is worth testing other discrimination techniques in future studies to improve the accuracy for white cultivars, such as the deep learning method, which has been shown to reliably classify plant species and diseases with both proximal and remote sensing hyperspectral data [72,73].

Our study demonstrated that the proximal spectroradiometer in the field with a solar radiation source is a practical method for predicting viral disease in grapevines. The spectral difference of diseased Pinot Noir showed a similar pattern to other GLD-infected red cultivars that were measured with an active sensor (leaf contacted), which demonstrated the reliability of the proximal sensing. The proximal sensing method collected data relatively simply and rapidly compared to on-leaf, contact-based sensors. The method developed from the present study could be readily applicable to different sensing platforms for future studies, such as a land vehicle platform for fast disease detection in vineyards [74], or using remote sensing platforms such as UAV or airplane platform for large-scale disease surveillance in vineyards. For example, MacDonald et al. [75] used remote-sensing hyperspectral images from an airplane platform for GLRaV-3 detection in Cabernet Sauvignon in the vineyards.

## 5. Conclusions

Detecting viral diseases in grapevines is achievable using a proximal hyperspectral sensor. The current study used a hand-held spectroradiometer to assess spectral differences between virus-infected and healthy vines under field conditions. The PLS-DA model was used to classify the diseased and healthy vines; this model proved to be a suitable method for viral disease detection in grapevines. The model result showed an optimal time window for detecting viruses in Pinot Noir and Chardonnay. Comparing the spectral reflectance between healthy and diseased vines, the red-edge spectral region was found to be an important region for GLD symptom detection in both Pinot Noir and Chardonnay. The 550 nm spectral region is important, particularly in red cultivars. This indicates that a multispectral sensor with green and red-edge bands could be used for viral disease detection in red cultivars. The proximal sensing method used in this study applies to other platforms such as ground-based vehicles or low-altitude remote-sensing platforms. Although the detection of viral diseases in white-berried grape cultivars remains challenging, this may be addressed in future studies through advances in sensing technology with a wider range of spectra and data processing methods and algorithms.

## Figures and Tables

**Table 1:** ELISA test results for both cultivars.

	Diseased (GLRaV-1 + GVA)	Healthy	Total
Chardonnay	134	40	174
Pinot Noir	72	101	173

**Table 2:** PLS-DA results for Chardonnay. Sample number: Disease = 134, Healthy = 40.

			Calibration Model Results						Cross-Validation Results					
			Confusion Matrix						Confusion Matrix					
Time	LVs	Predicted	Actual Disease	Actual Healthy	Sensitivity	F1-Score	Accuracy	MCC	Actual Disease	Actual Healthy	Sensitivity	F1-Score	Accuracy	MCC
Nov	2	Disease	80	15	0.60	0.70	0.60	0.19	77	14	0.57	0.68	0.59	0.19
		Healthy	54	25	0.63	0.42			57	26	0.65	0.42		
Dec	3	Disease	95	12	0.71	0.79	0.71	0.35	92	13	0.69	0.77	0.68	0.31
		Healthy	39	28	0.70	0.52			42	27	0.68	0.50		
Jan	3	Disease	96	10	0.72	0.80	0.72	0.40	91	15	0.68	0.76	0.67	0.26
		Healthy	38	30	0.75	0.56			43	25	0.63	0.46		
Feb	3	Disease	98	11	0.73	0.81	0.73	0.40	96	16	0.72	0.78	0.69	0.28
		Healthy	36	29	0.73	0.55			38	24	0.60	0.47		
Mar	4	Disease	102	9	0.76	0.83	0.76	0.47	99	11	0.74	0.81	0.74	0.41
		Healthy	32	31	0.78	0.60			35	29	0.73	0.56		
Apr	4	Disease	88	11	0.66	0.76	0.67	0.32	86	15	0.64	0.73	0.64	0.22
		Healthy	46	28	0.72	0.50			48	24	0.62	0.43		

**Table 3:** PLS-DA results for Pinot Noir. Sample number: Disease = 72, Healthy = 101.

			Calibration Model Results						Cross-Validation Results					
			Confusion Matrix						Confusion Matrix					
Time	LVs	Predicted	Actual Disease	Actual Healthy	Sensitivity	F1-Score	Accuracy	MCC	Actual Disease	Actual Healthy	Sensitivity	F1-Score	Accuracy	MCC
Nov	1	Disease	43	40	0.60	0.55	0.60	0.20	43	46	0.60	0.53	0.57	0.14
		Healthy	29	61	0.60	0.64			29	55	0.54	0.59		
Dec	3	Disease	48	33	0.68	0.63	0.67	0.35	45	34	0.63	0.60	0.65	0.29
		Healthy	23	68	0.67	0.71			26	67	0.66	0.69		
Jan	2	Disease	63	10	0.88	0.87	0.89	0.78	64	12	0.89	0.86	0.88	0.77
		Healthy	9	91	0.90	0.91			8	89	0.88	0.90		
Feb	2	Disease	61	3	0.85	0.90	0.92	0.84	58	2	0.81	0.88	0.91	0.81
		Healthy	11	98	0.97	0.93			14	99	0.98	0.93		
Mar	3	Disease	54	9	0.75	0.80	0.84	0.68	56	12	0.78	0.80	0.84	0.67
		Healthy	18	92	0.91	0.87			16	89	0.88	0.86		
Apr	5	Disease	66	1	0.92	0.95	0.96	0.92	66	3	0.92	0.94	0.95	0.89
		Healthy	6	100	0.99	0.97			6	98	0.97	0.96		

## Data Availability

Not applicable.
